# Development and Validation of a Predictive Model for Activities of Daily Living Dysfunction in Older Adults: Retrospective Analysis of Data From the China Health and Retirement Longitudinal Study

**DOI:** 10.2196/73030

**Published:** 2025-06-19

**Authors:** Fangbo Lin, Chao Liu, Hua Liu

**Affiliations:** 1 Rehabilitation Medicine Department, The Affiliated Changsha Hospital of Xiangya School of Medicine, Central South University, No. 311 Yinpan Road, Hunan Province, Changsha, 410008, China, 86 15111271991; 2 Neurology Department, Fujian Medical University Union Hospital, Fuzhou, China

**Keywords:** activities of daily living, elderly, risk prediction, nomogram model, frailty, CHARLS, China Health and Retirement Longitudinal Study

## Abstract

**Background:**

The global aging crisis has precipitated significant public health challenges, including rising chronic diseases, economic burdens, and labor shortages, particularly in China. Activities of daily living (ADL) dysfunction, affecting over 40 million Chinese older adults (16% of the aging population), severely compromises independence and quality of life while increasing health care costs and mortality. ADL dysfunction encompasses both basic ADL (BADL) and instrumental ADL (IADL), which assess fundamental self-care and complex environmental interactions, respectively. With projections indicating 65 million cases by 2030, there is an urgent need for tools to predict ADL impairment and enable early interventions.

**Objective:**

This study aimed to develop and validate a predictive nomogram model for ADL dysfunction in older adults using nationally representative data from the China Health and Retirement Longitudinal Study (CHARLS). The model seeks to integrate key risk factors into an accessible clinical tool to facilitate early identification of high-risk populations, guiding targeted health care strategies and resource allocation.

**Methods:**

A retrospective analysis was conducted on 5081 CHARLS wave 3 participants (2015‐2016) aged 60‐80 years. Participants were categorized into ADL dysfunction (n=1743) or normal (n=3338) groups based on BADL and IADL assessments. Forty-six variables spanning demographics, health status, biomeasures, and lifestyle were analyzed. After addressing missing data via multiple imputation, Least Absolute Shrinkage and Selection Operator (LASSO) regression and multivariable logistic regression identified 6 predictors. Model performance was evaluated using receiver operating characteristic curves, calibration plots, decision curve analysis, and Shapley additive explanations (SHAP) for interpretability.

**Results:**

The final model incorporated 6 predictors: the 10-item Center for Epidemiologic Studies Depression Scale depression score, number of painful areas, left-hand grip strength, 2.5-m walking time, weight, and cystatin C level. The nomogram demonstrated robust discriminative power, with area under the curve values of 0.77 (95% CI 0.76‐0.79) in both the training and testing sets. Calibration curves confirmed strong agreement between predicted and observed outcomes, while decision curve analysis highlighted superior clinical use over “treat-all” or “treat-none” approaches. SHAP analysis revealed depressive symptoms and physical frailty markers (eg, slow walking speed and low grip strength) as dominant predictors, aligning with existing evidence on ADL decline mechanisms.

**Conclusions:**

This study presents a validated nomogram for predicting ADL dysfunction in older adult populations, combining psychological, physical, and biochemical markers. The tool enables risk stratification, supports personalized interventions, and addresses gaps in geriatric care by emphasizing modifiable factors like pain management, depression, and mobility training. Despite limitations such as regional data biases and the retrospective design, the model offers scalable clinical value. Future research should incorporate social, environmental, and cognitive factors to enhance precision and generalizability.

## Introduction

The aging of the population, which has led to an increase in chronic diseases, financial burdens, and labor shortages, has become a significant public health concern and poses a major challenge to public health systems in China [1]. Aging refers to the gradual process of growing older, involving a series of changes over time, including physical, mental, and social transformations [2]. Each year, approximately 10% of older adults who were previously not disabled develop dysfunction in activities of daily living (ADL) [3]. A previous study estimated that more than 40 million older adults in China, or over 16% of the older adult population, currently experience ADL dysfunction. This number is expected to rise to 65 million by 2030 [4]. ADL dysfunction significantly affects the quality of life of older adults and is associated with increased health care expenditures, higher rates of institutionalization, and elevated mortality rates [5-7]. For many older adults, maintaining their independence for as long as possible is a key priority.

ADL dysfunction is typically assessed using the basic activities of daily living (BADL) and instrumental activities of daily living (IADL) scales. The BADL scale, which is widely used, assesses fundamental self-care abilities such as bathing, dressing, eating, and engaging in indoor activities [8]. To evaluate more complex aspects of daily living, Lawton and Brody [9] developed the IADL scale, which measures an individual’s ability to perform tasks that require more interaction with the environment, such as making telephone calls, shopping, cooking, and doing household chores. Given the strong correlation between BADL and IADL scores and overall functional ability [10], it is crucial to identify the factors contributing to ADL dysfunction in the older adults and to understand the characteristics of those affected.

A nomogram is a precise graphical representation of a predictive model based on regression analysis. It integrates multiple variables and visually presents the estimated probability of an event occurring. In this study, we developed and validated a comprehensive predictive model for assessing the risk of ADL dysfunction among older adults, using extensive data from the China Health and Retirement Longitudinal Survey (CHARLS). Subsequently, we created a nomogram that incorporates all selected risk factors, enabling rapid identification of older adults at risk of ADL dysfunction. The establishment of this predictive tool is expected to facilitate early detection, allowing for timely interventions that may reduce the incidence of ADL dysfunction in this population.

## Methods

### Study Population

This study is a retrospective study that used survey data from CHARLS wave 3, which were collected between August 2015 and January 2016. A total of 5081 samples were selected based on inclusion and exclusion criteria. Participants were included if they were aged between 60 and 80 years and were not missing data for essential variables. Exclusion criteria included individuals who were unwilling or unable to complete the survey. After applying these criteria, a total of 5081 participants were included in the analysis. Using the BADL and IADL assessment scales, participants were categorized based on their functional abilities. The BADL scale includes 6 basic tasks: dressing, bathing, eating, getting in and out of bed, using the toilet, and controlling urination and defecation. The IADL scale measures more complex activities, such as housework, food preparation, shopping, financial management, and medication adherence. Individuals with 1 or more disabilities in either the BADL or IADL domains were classified as having ADL dysfunction, while those without disabilities in either domain were classified as having normal ADL. Based on this classification, the participants were divided into 2 groups: the ADL normal group (n=3338) and the ADL dysfunction group (n=1743).

### Candidate Predictor Variables

Informed by clinical experience and previous studies [11,12], we analyzed 46 potential variables that might be associated with the risk of ADL dysfunction. These variables spanned various domains, including demographic characteristics, health status, lifestyle factors, biochemical indicators, and functional status. The variables selected for inclusion in this study are as follows: sex (1=male and 0=female), age, marital status (1=married and 0=unmarried), disability status (1=disability and 0=nondisability), presence of chronic disease (1=yes and 0=no), hypertension (1=yes and 0=no), diabetes mellitus (1=yes and 0=no), alcohol consumption (1=yes and 0=no), smoking status (1=yes and 0=no), history of falls (1=yes and 0=no), hip fracture (1=yes and 0=no), walking time (measured by the time required to walk 2.5 m), systolic and diastolic blood pressure, pulse rate, left and right hand grip strength, height, weight, waist circumference, BMI, respiratory function, the 10-item Center for Epidemiologic Studies Depression Scale (CESD-10), number of painful areas (eg, headache, shoulder pain, back pain, leg pain, and so on), sleep duration, physical activity (eg, visiting friends, playing games, and participating in community activities), blood test parameters (eg, white blood cell count, red blood cell volume, platelet count, glucose, and cholesterol levels), and several measures of social and cognitive factors (eg, educational level, sociality, and the triglyceride-glucose index. These variables were included for their potential relevance to ADL dysfunction.

### Statistical Analysis

Statistical analyses and figure generation were performed using R software (R Foundation for Statistical Computing). For variables with >20% missing data, those samples were excluded from the analysis. For variables with <20% missing data, the Multivariate Imputation by Chained Equations package was used to perform 5-fold imputation, and the imputed data most consistent with the sample trends were selected. Categorical variables were summarized using frequencies and percentages, while continuous variables were described by means and SDs. Differences between groups were assessed using *t* tests, chi-square tests, and nonparametric tests as appropriate. Nonparametric tests were used when data did not meet the assumptions for parametric testing, ensuring robustness and reliability of the analysis.

The data were split into training (n=3048) and testing (n=2033) sets using a 6:4 ratio. The training set was used to identify key predictors for ADL dysfunction. Initially, the Least Absolute Shrinkage and Selection Operator (LASSO) regression was applied to identify potential risk factors, minimizing multicollinearity and selecting the most predictive factors. Subsequently, a 10-fold cross-validation procedure was performed to determine the optimal tuning parameter (λ) for LASSO regression. Predictors selected by LASSO were then used in multivariate logistic regression, and a nomogram was constructed to visualize the influence of these predictors on the risk of ADL dysfunction. To simplify the model, predictors with minimal influence were discarded, and the remaining predictors were reanalyzed using multivariate logistic regression, followed by the construction of a final nomogram.

To evaluate the predictive performance of the model, several statistical tools were used, including receiver operating characteristic (ROC) curves, area under the curve (AUC), calibration plots, and decision curve analysis (DCA). ROC curves were used to assess the trade-off between sensitivity and specificity, while AUC quantified the model’s overall discriminatory power. Calibration plots evaluated the agreement between predicted probabilities and actual outcomes, ensuring the model’s reliability. DCA assessed the clinical use of the model by comparing the net benefits of true positive predictions against the harms of false positives and negatives. These comprehensive evaluation methods provided a assessment of the model’s accuracy and potential clinical impact.

Finally, to further explain the model’s feature variables, Shapley additive explanations (SHAP) values were calculated for each feature, and the predictive importance of these variables was visually represented through global importance plots, swarm plots, waterfall plots, and force plots. These visualizations facilitated a better understanding of how individual features contributed to the model’s predictions.

### Ethical Considerations

This retrospective study used anonymized data from wave 3 of the CHARLS database, which was ethically approved in June 2008 by the Biomedical Ethics Review Committee of Peking University (IRB00001052–11015) [13]. The individual data were anonymized before the study. In this study, patients and the public were not involved in the design, conduct, reporting, or dissemination plans of the research. All participants provided informed consent, and the study adheres to the ethical principles outlined in the Declaration of Helsinki.

## Results

### Flowchart

**Figure 1.**
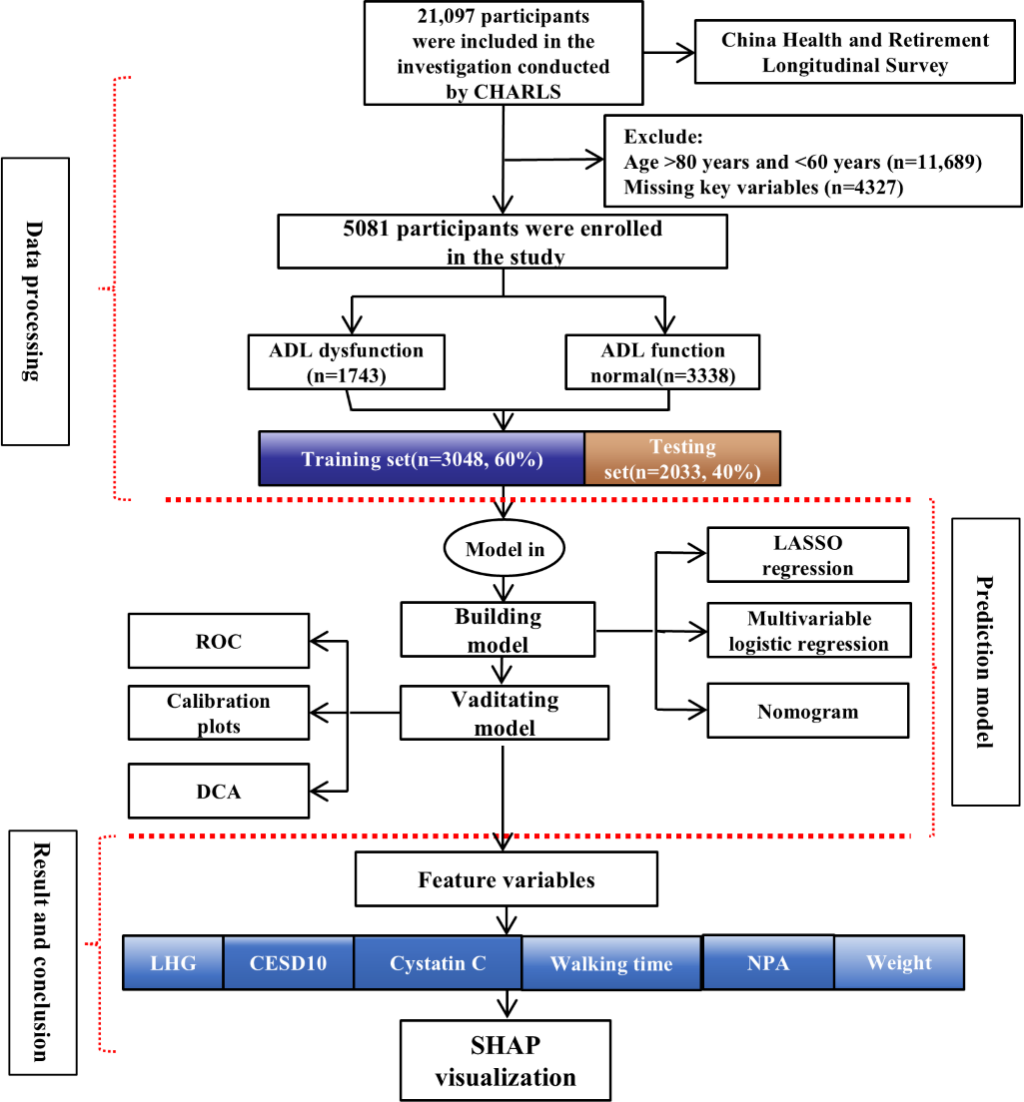
Flowchart of study cohort selection from CHARLS wave 3 (2015‐2016) for developing the activities-of-daily-living dysfunction prediction model. ADL: activities of daily living; CESD-10: 10-item Center for Epidemiologic Studies Depression Scale; CHARLS: China Health and Retirement Longitudinal Survey; DCA: decision curve analysis; LASSO: Least Absolute Shrinkage and Selection Operator; LHG: left grip strength; NPA: number of pain areas; ROC: receiver operating characteristic curve; SHAP: Shapley additive explanations.

The study flowchart is presented in Figure 1.

This retrospective cohort analysis of 5081 older adults (aged 60‐80 years old) across China evaluated inclusion and exclusion criteria and data imputation methods for developing a predictive nomogram. Participants were classified into ADL normal (no BADL/IADL disability) and ADL dysfunction (≥1 disability) groups using validated functional assessment scales.

### Baseline Characteristics

The study sample comprised 5081 participants, with an average age of 67 years, and a male-to-female ratio of 1.03. Of these, 3338 participants were classified into the ADL normal group, and 1743 participants were classified into the ADL dysfunction group. Significant clinical and sociodemographic differences were observed between the 2 groups, as summarized in Table 1. Key variables with significant differences (*P*<.05) include marital status, disability status, chronic diseases, hypertension, diabetes mellitus, alcohol consumption, smoking status, history of falls, hip fractures, walking time, systolic blood pressure, pulse rate, left and right hand grip strength, height, weight, waist circumference, respiratory function, depression (CESD-10), number of painful areas, sleep duration, physical activity, mean red blood cell volume, total cholesterol, C-reactive protein, glycosylated hemoglobin, uric acid, hematocrit, hemoglobin, cystatin C, sociality, and educational level.

In the comparative analysis of 5081 participants aged 60‐80 years old from the CHARLS, participants were stratified into the ADL normal (n=3338) and ADL dysfunction (n=1743) groups. Variables included demographics (age, sex, and marital status), comorbidities (hypertension, diabetes, and chronic diseases), lifestyle factors (smoking and alcohol use), biomeasures (grip strength, walking time, and cystatin C), and functional parameters. Significant differences (*P*<.05) between groups were highlighted, reflecting associations with ADL impairment in Chinese older adults.

**Table 1.** Baseline sociodemographic and clinical characteristics of older adults (ADL^a^ normal group vs ADL dysfunction group) in the China Health and Retirement Longitudinal Survey wave 3 (China, 2015‐2016).

Characteristics	ADL function normal group (n=3338)	ADL dysfunction group (n=1743)	*P* value
Gender, n (%)			<.001
Male	1865 (55.9)	713 (40.9)	
Female	1473 (44.1)	1030 (59.1)	
Marital stauts, n (%)			<.001
Yes	2814 (84.3)	1391 (79.8)	
No	524 (15.7)	352 (20.2)	
Hypertension, n (%)			<.001
Yes	1220 (36.5)	808 (46.4)	
No	2118 (63.5)	935 (53.6)	
Diabetes, n (%)			<.001
Yes	338 (10.1)	260 (14.9)	
No	3000 (89.9)	1483 (85.1)	
Disability, n (%)			<.001
Yes	1033 (30.9)	946 (54.3)	
No	2305 (69.1)	797 (45.7)	
Drink, n (%)			<.001
Yes	1669 (50.0)	774 (44.4)	
No	1669 (50.0)	969 (55.6)	
Smoke, n (%)			<.001
Yes	1711 (51.3)	730 (41.9)	
No	1627 (48.7)	1013 (58.1)	
Fall, n (%)			<.001
Yes	489 (14.6)	503 (28.9)	
No	2849 (85.4)	1240 (71.1)	
Hip fracture, n (%)			<.001
Yes	47 (1.4)	64 (3.7)	
No	3291 (98.6)	1679 (96.3)	
Chronic, n (%)			<.001
Yes	2668 (79.9)	1593 (91.4)	
No	670 (20.3)	150 (8.6)	
Walking time (second), mean (SD)	3.19 (0.95)	3.73 (1.48)	<.001
Systolic pressure (mm Hg), mean (SD)	130.12 (19.96)	131.62 (20.59)	.01
Diastolic pressure (mm Hg), mean (SD)	74.59 (11.56)	74.58 (11.46)	.96
Pulse (beats per min), mean (SD)	73.30 (10.7)	74.26 (11.63)	.003
Left hand grip (N), mean (SD)	28.62 (9.16)	23.74 (9.52)	<.001
Right hand grip (N), mean (SD)	29.82 (9.71)	24.98 (9.57)	<.001
Height (m), mean (SD)	1.58 (0.09)	1.55 (0.1)	<.001
Weight (Kg), mean (SD)	59.05 (11.81)	58.04 (11.53)	.003
Waist (cm), mean (SD)	84.54 (13.47)	85.37 (14.85)	.04
BMI (kg/m^2^), mean (SD)	24.12 (18.48)	24.44 (11.62)	.50
Respiratory test value (ml), mean (SD)	308.28 (120.26)	266.65 (105.92)	<.001
Self-rated depression scale (score), mean (SD)	6.50 (5.35)	11.52 (6.99)	<.001
Number of painful areas (score), mean (SD)	0.93 (2.38)	3.41 (4.35)	<.001
Sleep hours, mean (SD)	6.49 (1.9)	5.86 (2.2)	<.001
Activity (score), mean (SD)	0.87 (1.03)	0.74 (0.92)	<.001
White blood cell (10^12/L), mean (SD)	5.92 (1.76)	6.07 (1.91)	.006
Mean red blood cell volume (fl), mean (SD)	92.45 (7.51)	91.73 (8.14)	.002
Platelet (10^9/L), mean (SD)	198.09 (72.49)	201.84 (77.99)	.09
Blood urea nitrogen (mg/dl), mean (SD)	16.04 (4.62)	16.28 (5.06)	.08
Glucose (mg/dl), mean (SD)	104.23 (33.34)	104.74 (37.14)	.62
Blood creatinine (mg/dl), mean (SD)	0.84 (0.25)	0.84 (0.39)	.98
Total cholesterol (mg/dl), mean (SD)	184.37 (35.76)	187.06 (37.53)	.01
Triglyceride (mg/dl), mean (SD)	135.64 (84.62)	140.27 (87.66)	.07
High density cholesterol (mg/dl), mean (SD)	51.58 (12.01)	51.99 (12.29)	.25
Low density cholesterol (mg/dl), mean (SD)	103.30 (28.60)	104.38 (29.43)	.20
C-reactive protein (mg/l), mean (SD)	2.62 (4.72)	3.14 (7.08)	.002
Glycosylated hemoglobin (%), mean (SD)	6.03 (0.96)	6.12 (1.11)	.002
Uric acid (mg/dl), mean (SD)	5.08 (1.39)	4.93 (1.44)	<.001
Hematocrit (%), mean (SD)	41.73 (5.34)	40.72 (5.52)	<.001
Hemoglobin (g/dl), mean (SD)	13.75 (1.80)	13.42 (1.85)	<.001
Cystatin C (mg/l), mean (SD)	0.89 (0.2)	0.93 (0.31)	<.001
Sociality (score), mean (SD)	0.91 (1.11)	0.76 (0.95)	<.001
Age (years), mean (SD)	66.77 (5.17)	67.73 (5.41)	<.001
Educational level, mean (SD)	1.87 (0.99)	1.54 (0.82)	<.001
Triglyceride-glucose index, mean (SD)	8.68 (0.62)	8.72 (0.63)	.07
Triglyceride glucose–BMI, mean (SD)	210.02 (155.18)	213.70 (99.89)	.36

a ADL: activities of daily living.

### Development of the Predictive Nomogram

**Figure 2.**
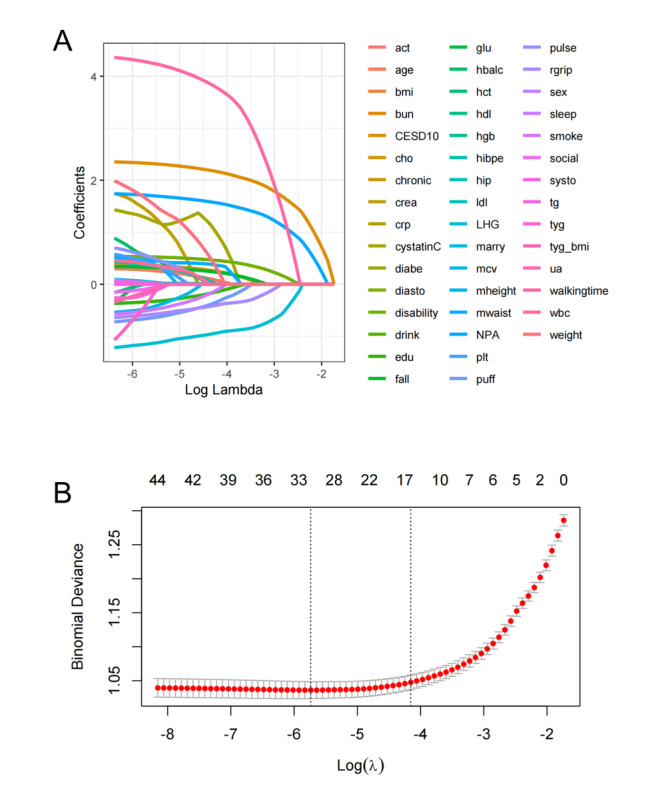
Least Absolute Shrinkage and Selection Operator regression feature selection for ADL dysfunction predictors in China Health and Retirement Longitudinal Survey (2015‐2016): (A) Coefficient profiles of 46 candidate variables analyzed via Least Absolute Shrinkage and Selection Operator regression to identify predictors of ADL dysfunction in Chinese older adults and (B) 10-fold cross-validation for optimal lambda (λ) selection, minimizing partial likelihood deviance. Features with nonzero coefficients (eg, Center for Epidemiologic Studies Depression Scale, walking time, and cystatin C) were retained for nomogram development. ADL: activities of daily living; CESD10: 10-item Center for Epidemiologic Studies Depression Scale; LHG: left grip strength.

**Figure 3.**
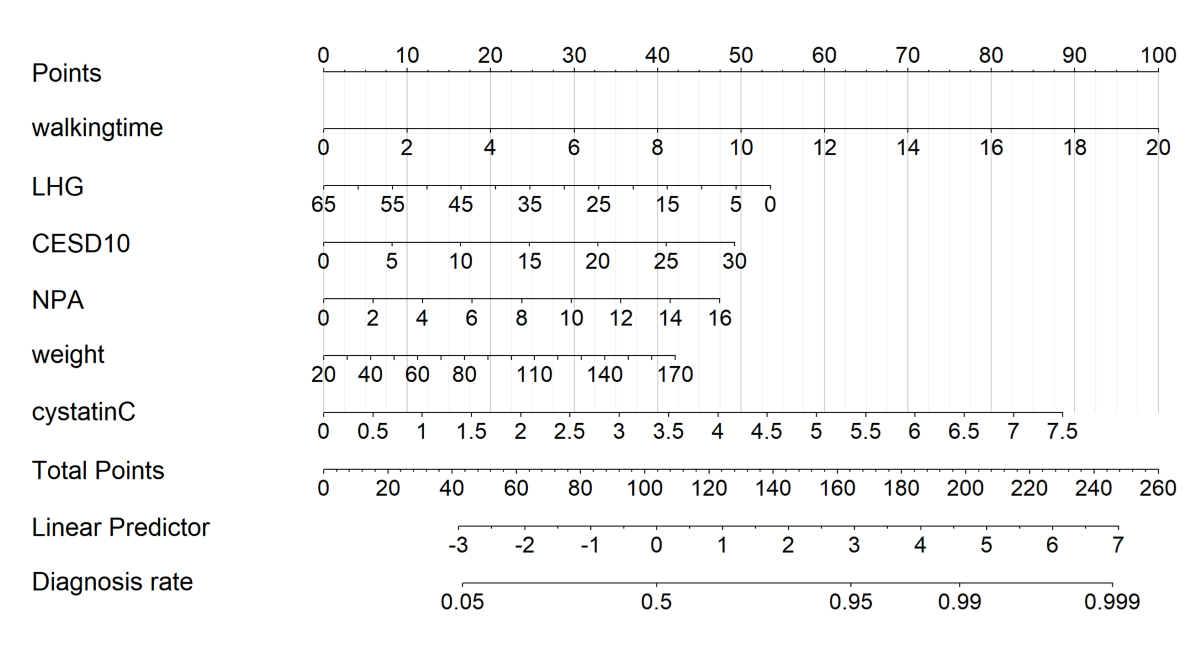
Nomogram for predicting activities-of-daily-living dysfunction risk in Chinese older adults (China Health and Retirement Longitudinal Survey, 2015‐2016). Graphical tool integrating six predictors (Center for Epidemiologic Studies Depression Scale, pain areas, left grip strength, 2.5-m walking time, weight, and cystatin C) to estimate individualized risk of activities-of-daily-living dysfunction. Scores for each variable are summed to calculate total points, corresponding to predicted probability on a 0%‐100% scale. Derived from 3048 training set participants in the China Health and Retirement Longitudinal Survey cohort. CESD10: 10-item Center for Epidemiologic Studies Depression Scale; LHG: left grip strength; NPA: number of pain areas; walkingtime: 2.5-meter walking time.

Participants were randomly divided into a training set (n=3048) and a testing set (n=2033) in a 6:4 ratio. LASSO regression was applied to identify the most important predictors of ADL dysfunction. After performing 10-fold cross-validation, predictors with nonzero coefficients were selected (Figures 2A and 2B). These predictors were then incorporated into a multivariate logistic regression model, factors with *P* values>.05 (eg, respiratory function and sleep duration) were removed in the multivariate logistic regression. Ultimately, 6 independent predictors were retained: 2.5-m walking time, left hand grip strength, CESD-10 score, number of painful areas, weight, and cystatin C. Based on these predictors, a nomogram was constructed to estimate the risk of ADL dysfunction in the older adults. The nomogram visually represents the contribution of each factor to the likelihood of dysfunction, with the bottom scale indicating the predicted probability. Higher scores correspond to a greater risk of ADL dysfunction (Figure 3).

### Performance and Clinical Application of the Nomogram

**Figure 4.**
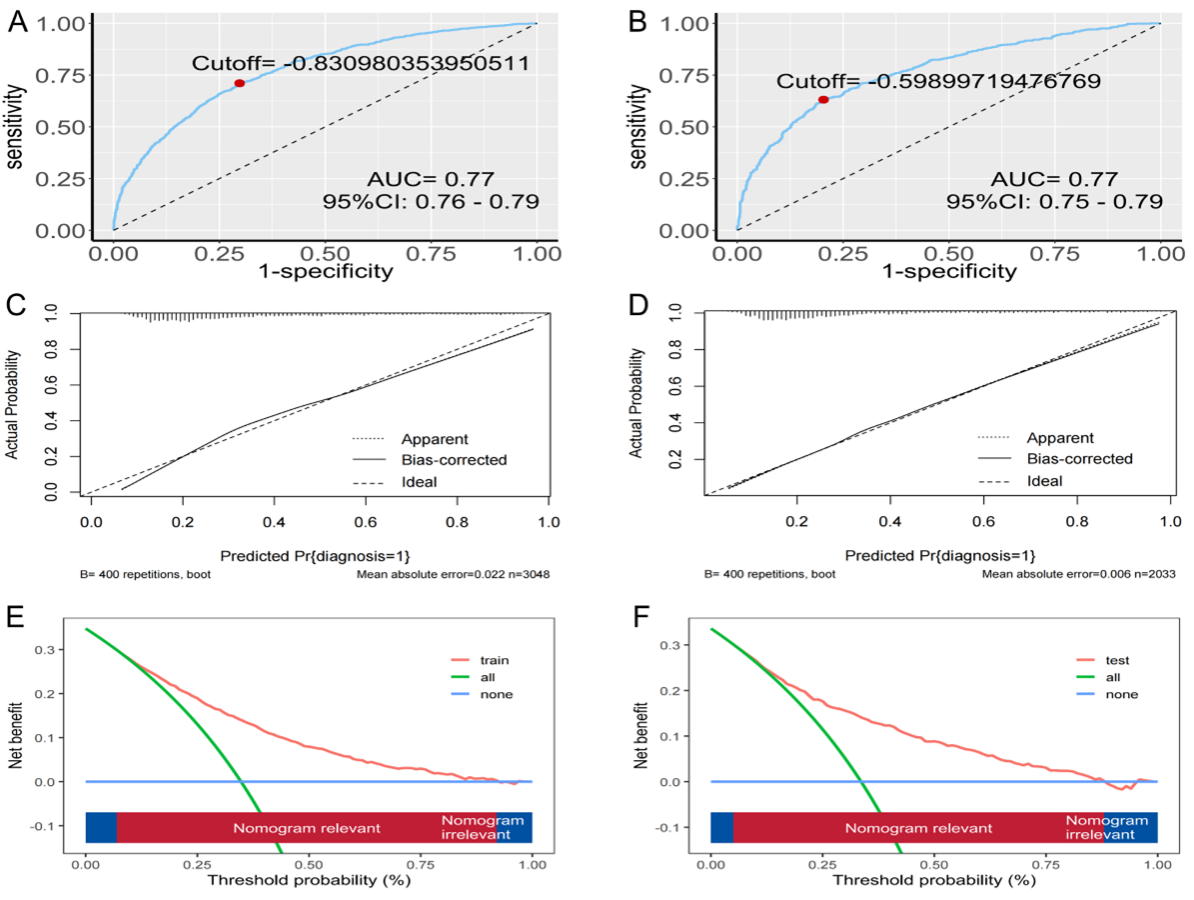
Predictive performance and clinical use of the activities-of-daily-life dysfunction nomogram: (A-B) receiver operating characteristic curves showing discriminative power (AUC 0.77, 95% CI 0.76‐0.79) for training (A) and testing (B) sets in China Health and Retirement Longitudinal Survey (2015‐2016); (C-D) calibration plots demonstrating agreement between predicted and observed activities-of-daily-living dysfunction probabilities; and (E-F) decision curve analysis comparing net clinical benefits of the nomogram against “treat-all” and “treat-none” strategies in Chinese older adults. AUC: area under the curve.

To evaluate the performance of the nomogram, the AUC values were calculated. In the training set, the AUC was 0.77 (95% CI 0.76‐0.79), and in the testing set, the AUC was 0.77 (95% CI 0.75‐0.79), demonstrating the model’s high predictive accuracy (Figures 4A and 4B).

The nomogram’s calibration curves show agreement between the predicted and observed probabilities in both the training and testing sets, further confirming the model’s accuracy and reliability (Figures 4C and 4D).

In addition, DCA was used to assess the clinical use of the model. The results indicated that the model’s net benefit in the internal validation set was better than both the “treat-all” and “treat-none” scenarios, highlighting its clinical use and predictive performance (Figures 4E and 4F).

### Explanation of Model Characteristic Variables

**Figure 5.**
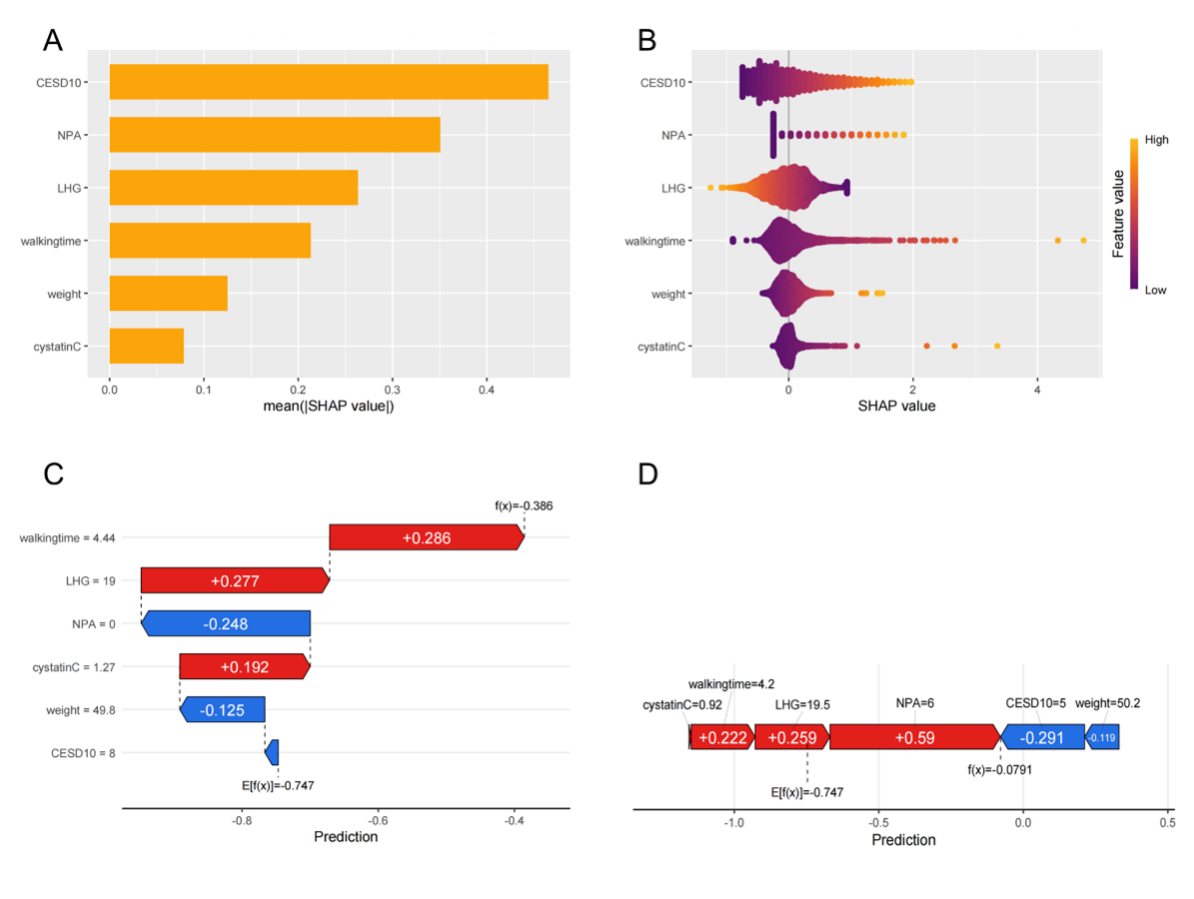
Interpretability of the activities-of-daily-life dysfunction prediction model using Shapley additive explanations values: (A) Global Shapley additive explanations importance plot ranking features by contribution to activities-of-daily-living dysfunction predictions in China Health and Retirement Longitudinal Survey (2015‐2016, n=5081); (B) swarm plot depicting directionality (positive/negative) of predictors (eg, Center for Epidemiologic Studies Depression Scale, pain areas); and (C-D) waterfall and force plots illustrating individualized risk contributions for 2 participants (China Health and Retirement Longitudinal Survey ID: Sample A vs B), highlighting variable-specific impacts on model output. CESD10: 10-item Center for Epidemiologic Studies Depression Scale; LHG: left grip strength; NPA: number of pain areas; walkingtime: 2.5-meter walking time.

SHAP values were calculated to assess the influence of each characteristic variable in the model. The global importance plot and swarm plot (Figures 5A and 5B) reveal the relative importance of the 6 key variables in predicting ADL dysfunction, ranked from highest to lowest: CESD-10 score, number of painful areas, left hand grip strength, 2.5-m walking time, weight, and cystatin C. Except for left hand grip strength, all other variables were positively correlated with ADL dysfunction.

To illustrate the individual contributions of these variables, 2 random samples were selected, and the predictive effects of the variables were visualized through waterfall and force plots (Figures 5C and 5D). These plots demonstrate the distinct effects of each factor on ADL dysfunction for individual participants, providing valuable insights into the practical significance of the model’s predictors.

## Discussion

### Principal Findings

This study identified 6 robust predictors of ADL dysfunction in the older adults, namely CESD-10 depression score, number of painful areas, left-hand grip strength, 2.5-m walking time, weight, and cystatin C levels. These factors reflect multidimensional contributions from psychosocial states, physical frailty markers, systemic inflammation, and biomechanical stressors. Notably, participants with a history of disability, chronic diseases, or falls exhibited significantly higher ADL dysfunction risk, consistent with previous evidence on functional decline pathways [14,15]. The 2.5-m walking time defined in this study is negatively correlated with gait speed. Key findings further highlighted walking speed as a critical functional indicator. Slow gait (≥3.73 seconds for 2.5 m) correlated strongly with ADL limitations, echoing its status as a “sixth vital sign” for aging populations [16-19]. Similarly, left-hand grip strength emerged as a stronger predictor than right-hand strength, suggesting asymmetric hand function may play underrecognized roles in daily task performance. Depressive symptoms (CESD-10>11.5) and polyarticular pain (>3 body areas) were dominant risk amplifiers, aligning with their bidirectional relationships with physical disability [20-22].

### Comparison With Previous Work

While many studies have identified predictors of ADL dysfunction in community-dwelling older adults, research constructing ADL dysfunction prediction models is rare, with only a few reporting ADL dysfunction prediction models. Jonkman et al [23] developed and validated a 3-year ADL dysfunction prediction model for adults aged 65‐75 years, selecting 10 of 22 predictors, including physical performance indicators, age, BMI, depressive symptoms, and chronic diseases. Covinsky et al [24] constructed and validated an ADL dysfunction prediction model for community residents aged 70 years and older, including 9 predictors: age, comorbidities, cognitive function, low BMI, and 5 functional limitation indicators. Recent domestic studies have found that a nomogram incorporating 10 predictors age, education level, social activity frequency, drinking and smoking habits, smoking frequency, comorbidity condition, self-reported health status, gait speed, cognitive function, and depressive symptoms can effectively predict disability risks [25]. Our study aligns with previous research in focusing on depressive symptoms, weight, and gait speed as predictors. However, it differs by incorporating new indicators: number of painful body regions, left-hand grip strength, and cystatin C. Our findings reinforce established associations between depression, obesity, and ADL decline [21,26-28]. For instance, the link between CESD-10 scores and ADL aligns with Carrière et al’s [20] longitudinal data showing depression predicts subsequent functional dependence. Similarly, weight’s positive association with ADL dysfunction parallels studies identifying obesity-related metabolic strain and mobility limitations as key mechanisms [26,29]. However, our model extends existing literature in 3 ways. First, pain is a critical factor contributing to ADL dysfunction and poor functional health in older adults [30]. It is cross-sectionally associated with ADL function and predicts a decline in ADL functioning over time [31]. Older individuals often become less active due to pain, which leads to physical deconditioning and perpetuates a cycle of pain and activity restriction [32]. Second, cystatin C, a marker of renal function and inflammation emerged as a novel biochemical predictor, potentially reflecting subclinical frailty progression [33]. Cystatin C may reflect renal function decline, and renal dysfunction is associated with sarcopenia and cardiovascular events [34]. This may underlie its potential mechanism in predicting ADL dysfunction. Third, the left-hand grip strength’s predictive dominance contrasts with conventional bilateral grip assessments [35-37], suggesting laterality-specific strength may better capture real-world functional demands.

### Strengths and Limitations

The study’s strengths include the use of nationally representative CHARLS data with a large sample size, rigorous LASSO-multivariable regression for predictor selection, and validation through AUC (0.77), calibration, and SHAP interpretability. The integration of psychological, biomechanical, and biochemical indicators provides a holistic view of ADL risk. Importantly, these predictors are easily accessible and low-cost, enabling assessment for older adults in community hospital outpatient clinics and facilitating broad implementation.

Limitations warrant consideration. First, the CHARLS data, while valuable, may be limited by regional and cultural factors, which may affect the generalizability of our findings. Future research should aim to include more diverse populations. Second, the variables selected in this study may have excluded important factors such as social support, living environment, and cognitive function, which could reduce the model’s accuracy. Future research could incorporate these variables to enhance the model’s precision. Third, as a retrospective study, the reliance on self-reported data introduces the potential for information bias, particularly in the reporting of chronic diseases and falls. Future prospective studies are needed to track changes in these variables over time and improve the model’s predictive accuracy. Finally, grip strength data were not differentiated by dominant hand. Future studies could explore the potential impact of grip strength side on the results.

### Future Directions

Future studies should incorporate longitudinal designs to track dynamic interactions between cognitive decline, social support networks, and environmental factors in shaping ADL trajectories. The role of cystatin C as a biomarker warrants further investigation to elucidate its mechanistic links to frailty progression and chronic inflammation [33]. In addition, task-specific functional magnetic resonance imaging or observational studies could clarify the dominance of left-hand grip strength and refine asymmetry-targeted rehabilitation protocols. Comparative analyses of weight-adjusted indices versus traditional metrics like BMI may improve obesity-related risk stratification [29]. Finally, validation in multiethnic cohorts and real-world clinical settings is critical to ensure model generalizability and inform scalable interventions addressing modifiable psychosocial and physical risk factors.

### Conclusions

This tool is a predictive model developed to assess the risk of functional impairment in daily living among older adults. It incorporates factors such as the CESD-10, number of painful areas, left-hand grip strength, 2.5-m walking time, weight, and cystatin C. During both the development and validation phases, the model demonstrated discriminative ability and accuracy, effectively identifying individuals at higher risk and maintaining reliability when applied to new data.

For health care professionals, the model can assist in making more informed care decisions, help prioritize interventions, and support the customization of treatment plans for high-risk individuals. By identifying those at greater risk for functional decline, the model may contribute to improving the quality of life for older adults, enhancing their independence, and promoting overall well-being. In addition, it may aid in the more efficient allocation of health care resources by directing attention to individuals who would benefit most from targeted interventions.

## Abbreviations

ADL: activities of daily living
AUC: area under the curve
BADL: basic activities of daily living
CESD-10: 10-item Center for Epidemiologic Studies Depression Scale
CHARLS: China Health and Retirement Longitudinal Survey
DCA: decision curve analysis
IADL: instrumental activities of daily living
LASSO: Least Absolute Shrinkage and Selection Operator
ROC: receiver operating characteristic
SHAP: Shapley additive explanations
